# Next-Generation Fluorogen-Based Reporters and Biosensors for Advanced Bioimaging

**DOI:** 10.3390/ijms20246142

**Published:** 2019-12-05

**Authors:** Tiphaine Péresse, Arnaud Gautier

**Affiliations:** 1 Sorbonne Université, École Normale Supérieure, PSL University, CNRS, Laboratoire des Biomolécules, LBM, 75005 Paris, France; tiphaine.peresse@ens.fr; 2 Institut Universitaire de France (IUF), 1 rue Descartes, 75005 Paris, France

**Keywords:** fluorogenic systems, protein labeling, RNA labeling, advanced microscopy

## Abstract

Our ability to observe biochemical events with high spatial and temporal resolution is essential for understanding the functioning of living systems. Intrinsically fluorescent proteins such as the green fluorescent protein (GFP) have revolutionized the way biologists study cells and organisms. The fluorescence toolbox has been recently extended with new fluorescent reporters composed of a genetically encoded tag that binds endogenously present or exogenously applied fluorogenic chromophores (so-called fluorogens) and activates their fluorescence. This review presents the toolbox of fluorogen-based reporters and biosensors available to biologists. Various applications are detailed to illustrate the possible uses and opportunities offered by this new generation of fluorescent probes and sensors for advanced bioimaging.

## 1. Introduction

Our understanding of the inner workings of cells and organisms is inherently linked to our ability to visualize biochemical events with high spatial and temporal resolution. The introduction of fluorescent proteins (FP) as genetically encoded fluorophores has been essential in our quest of visualizing proteins and various biochemical activities in living cells. The reasons why biologists rapidly adopted fluorescent proteins such as the green fluorescent protein (GFP) resides in their straightforward use that allowed, with minimal expertise in molecular biology and fluorescence microscopy, to obtain scientifically meaningful images and information. Since the first use of GFP as genetically encoded fluorophore in 1994, FPs with various spectral properties have been discovered and developed for various applications [1,2,3,4]. GFP-like FPs have been used for the visualization of (fusion) proteins in various hosts and for the design of fluorescent biosensors that enable us to see various biomolecules and biochemical activities.

**Figure 1:**
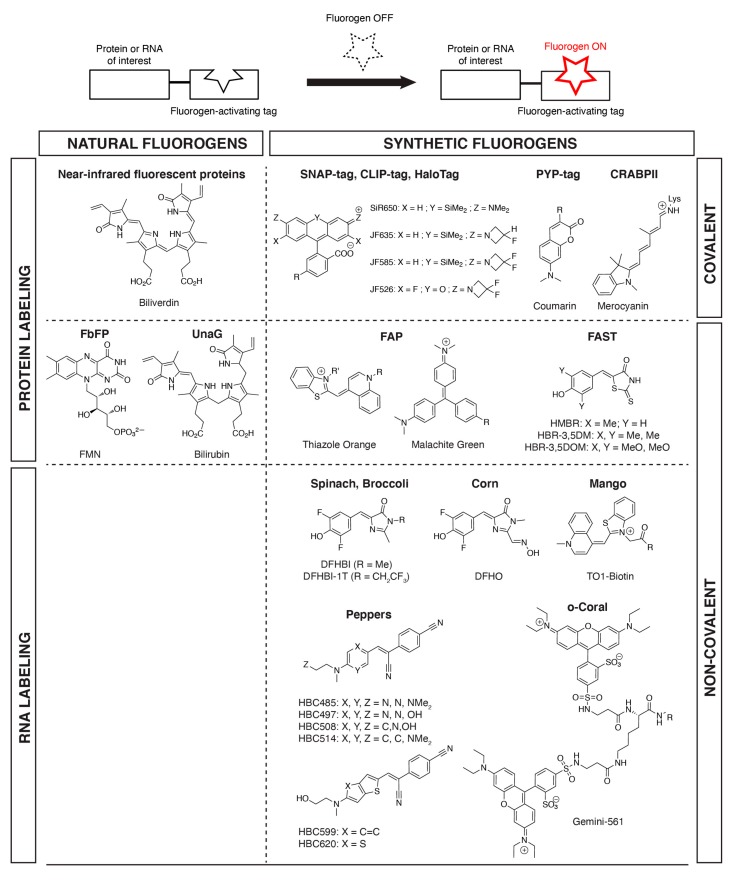
Examples of fluorogens found in fluorogen-based reporters and biosensors. Proteins and RNA of interest can be fluorescently labeled in a selective fashion through fusion to tags that (covalently or non-covalently) bind (natural or synthetic) fluorogens and activate their fluorescence.

Although GFP-like FPs have revolutionized our way to image biomolecules and biochemical activities, intrinsic shortcomings, such as their size and tendency to oligomerize, their need for molecular oxygen, their slow fluorophore maturation, and their restriction to a genetically encoded fluorophore, have motivated the development of alternative fluorescent reporters [1,5,6]. Among them, one can cite alternative FPs that incorporate endogenous chromophores naturally present in cells, and chemical–genetic reporters, which are semisynthetic hybrid systems composed of a genetically encoded tag and a synthetic chromophore (Figure 1). Engineered mostly from natural photoreceptors, natural chromophore-based FPs rely on the property of chromophores such as flavin, biliverdin, and bilirubin to strongly fluoresce when appropriately bound, and to be almost nonfluorescent when free, ensuring high contrast. The fact that such fluorogenic chromophores (hereafter called fluorogens) are naturally present in cells is a clear advantage of these reporters as additional fluorogen supply is a priori not necessary. Chemical–genetic reporters, on the other hand, are composed of a genetically encoded tag that specifically recognizes a synthetic chromophore exogenously applied. The use of synthetic fluorogens that light up upon reaction/interaction with the genetic tag allows high-contrast imaging, since free, unbound chromophores remain invisible. In terms of design, chemical-genetic reporters are conceptually similar to natural chromophore-based fluorescent proteins, as they both rely on a genetically encoded part (that allows absolute labeling selectivity) that recognizes, in a specific fashion, a fluorogen (natural or synthetic) that is bright only when bound, but is otherwise dark when free. One advantage of chemical–genetic reporters over natural chromophore-based FP is that natural chromophores are not hijacked from their physiological function, reducing the risk of perturbing cellular processes and inducing cellular stress. Moreover, the spectral and physicochemical properties of synthetic fluorogens can be tuned by using the power of modern chemistry; the additional labeling step provides opportunities to design innovative protocols for on-demand labeling, and the labeling of biomolecules other than proteins such as RNA can be envisioned. In this review, we present the design and applications of various fluorescent reporters based either on natural or synthetic fluorogens, highlighting in particular how they complement the FP toolbox and offer new opportunities for advanced imaging.

## 2. Fluorescent Reporters and Biosensors Based on Natural Fluorogenic Chromophores

Many chromophore-binding proteins, such as photoreceptors, are naturally found in various plant, algae, and bacteria species. Thanks to the progress in molecular biology, modifying the properties of proteins has become easier and easier, enabling photoreceptors to evolve into fluorescent proteins and optimize their photophysical and spectral features. The three main classes of fluorescent reporters engineered from natural photoreceptors bind the endogenous fluorogenic chromophores flavin, biliverdin, and bilirubin.

### 2.1. Flavin-Binding Cyan–Green Fluorescent Proteins

Flavin mononucleotide (FMN)-based fluorescent proteins (FbFPs) have been designed from photoactive light-oxygen and voltage-sensing domains (LOV) found in blue light photoreceptors of various plant, algae, and bacteria species [7,8,9]. In native LOV domains, blue light induces a reversible covalent bond between FMN and a conserved cysteine residue in the binding pocket, which leads to a change of conformation and thus a biological response. Cysteine replacement by alanine and directed evolution enabled to kill this natural photocycle and to generate FbFPs fluorescing cyan–green light, thanks to the noncovalently bound FMN (ubiquitously present in cells) [10]. This engineering strategy also allowed the development of miniSOG from the LOV2 domain of *Arabidopsis thaliana* phototropin-2, a system that not only fluoresces but also generates singlet oxygen upon blue light illumination [11].

The unique properties of FbFPs enabled it to overcome some limitations of GFP-like FPs [12]. FbFPs are, unlike GFP-like FPs, O_2_-independent, allowing experiments in anaerobic conditions [10,13]. These systems have proved to be suitable for monitoring intracellular processes and studying host–pathogen interactions in hypoxic conditions in various organisms (bacteria or fungi) and cultured mammalian cells [10,14,15,16,17,18,19]. As an example, FbFPs have been used as a reporter to study the human pathogen anaerobic bacteria *Clostridium difficile*, *Bacteroides fragilis*, and *Porphyromonas gingivalis*, as well as the extracellular secretion of proteins in *Clostridium difficile* [14,15,17]. A ratiometric Förster resonance energy transfer (FRET) oxygen sensor was developed by fusing FbFP to the yellow fluorescent protein (YFP). Since oxygen is essential for the fluorescence maturation of YFP (the FRET acceptor), but not for that of FbFP (the FRET donor), the FRET efficiency directly correlates with the oxygen concentration [20]. FbFPs were recently utilized for the design of other types of biosensors, such as pH FRET-based biosensor [21]. Another advantage of FbFPs resides in their small size, in average 12 to 16 kDa. A smaller tag means lower risks of generating dysfunctional protein fusions, and reduced genetic footprint, which was shown to be advantageous for virus labeling [12].

The FbFP MiniSOG also presents the ability to generate reactive oxygen species (ROS) upon excitation, which opened new opportunities for correlative light and electron microscopy (as it is fluorescent and allows local polymerization of diaminobenzidine into an osmiophilic product detectable by electron microscopy) and for applications such as local photooxidation, cell ablation, or chromophore-assisted light inactivation (CALI) [11]. Recent studies have shown that miniSOG and variants (such as SOPP2, SOPP3, and MiniSOG2) allowed effective optogenetic inactivation of antimicrobial agents and cell ablation of specific cell types (neuron, muscle, or epidermis) in *Caenorhabditis elegans* and *Drosophila* larva [22,23,24,25]. Very recently, a split version of miniSOG was designed to study protein–protein interactions by light microscopy and electron microscopy [26]. This split system allowed for the visualization of AP-1 transcriptional complex in nucleoli of mammalian cells.

### 2.2. Bilirubin-Binding Green Fluorescent Protein

A bilirubin-binding fluorescent protein, displaying similar excitation and emission wavelengths to GFP and FbFPs, was isolated from Japanese unagi eels and characterized by the group of Miyawaki [27]. The fluorescence properties of this monomeric protein, called UnaG, results from a bilirubin (BR) molecule tightly encased noncovalently within the protein cavity. Being an endogenous catabolic product of hemes, bilirubin is present in high concentrations in mammalian cells, allowing the direct formation of fluorescent complex. To use UnaG in species that do not synthetize bilirubin (e.g., bacteria), bilirubin can be added exogenously in culture media [27]. UnaG shares many similarities with FbFPs, such as a small size (15 kDa), an oxygen-independent fluorescence, and almost instantaneous fluorescence maturation upon bilirubin binding. The oxygen-independence feature has been exploited to visualize hypoxia in tumors at the cellular level [28]. UnaG has been reported to be brighter than FbFPs, making it one of the brightest alternatives to GFP [27]. 

UnaG has been further employed for the development of fluorescence bimolecular complementation assays and biosensors [29,30]. A split version of UnaG called uPPI (UnaG-based protein–protein interaction (PPI) reporter) was developed through a structure-guided approach. This split version has been validated with the FRB/FKBP system in mammalian cells. The addition of rapamycin, which induces the association of FRB and FKBP, allowed UnaG complementation in the presence of bilirubin [29]. Further engineering allowed the design of a UnaG-based calcium sensor by coupling UnaG with the calcium-binding protein calmodulin. This dual-ligand modulable fluorescent protein is able to bind both bilirubin (via UnaG) and calcium (via calmodulin). The binding of bilirubin is negatively regulated by the binding of calcium through conformational change, generating a fluorescence signal that depends on calcium concentrations [30]. 

### 2.3. Biliverdin-Binding Far-Red and Infrared Fluorescent Proteins

Fluorescent proteins emitting in the far-red and the infrared (IR) were engineered from bacterial phytochrome binding the fluorogenic biliverdin (BV). The first example of IR fluorescent proteins, IFP1.4, was developed by using the N-terminal of PAS and GAF domains from *Deinococcus radiodurans* phytochrome [31]. In the natural photoreceptor, BV is covalently attached via a thioether bound and undergoes a reversible *cis–trans* photoisomerization at its C15=C16 double bond under far-red light illumination. Mutations were introduced to prevent photoisomerization and conformationally lock BV, resulting in a drastic increasing of the fluorescence. A brighter version, namely IFP2.0, was further developed by protein engineering [32]. Although BV is ubiquitously present in all aerobic organisms, as it is an initial intermediate in heme catabolism by heme oxygenase (HO), external supply was needed to obtain optimal imaging. Alternatively, co-expression of HO was shown to boost intracellular BV levels, but with the disadvantage of risking the dysregulation of cellular homeostasis [32]. A truncating version of the bacteriophytochrome RpBphP2 from *Rhodopseudomonas palustris* has proved to be an improved near-infrared fluorescent protein version (iRFP) [33]. Due to its higher affinity for BV, iRFP does not require an additional exogenous supply of BV or co-expression of HO; iRFP variants were engineered to expand the color range available from 670 to 720 nm [34]. 

The iRFPs are dimeric proteins, and IFP2.0 tends to form dimers at high concentrations [35]. As these dimeric forms represent an issue in bioimaging, as they may disrupt the function of the tagged protein and can lead to mislocalization and protein aggregation, a fully monomeric version called mIFP was engineered from a monomeric truncated bacteriophytochrome from *Bradyrhizobium* [35]. As mIFP displays weak brightness due to a low affinity with BV, brighter monomeric iRFPs (miRFPs) were next engineered from bacteriophytochrome RpBphP1 with BV specificity and affinity similar to those of the dimeric forms [36]. These monomeric versions are called miRFP670, miRFP703, miRFP709, and miRFP720, and they are the most red-shifted monomeric near-infrared (NIR) fluorescent proteins [37].

Recently, a near-infrared fluorescent protein was engineered from the cyanobacteriochrome allophycocyanin (APC) of *Cyanobacterium trichodesmium* [38]. Naturally, APC binds phycocyanobilin (PCB). Random mutagenesis and directed evolution have been necessary to generate the small ultra-red fluorescent protein smURFP, a NIR FP which covalently binds BV instead of PCB [38]. This homodimeric protein (composed of two 15 kDa subunits) is one of the brightest far-red/near-infrared fluorescent proteins reported, but it requires the supply of exogenous BV or the more cell-permeant biliverdin methyl ester, for optimal fluorescence in cells [39]. To address issues due to dimer formation or low BV binding constant, a small monomeric protein (15 kDa) called miRFP670nano was recently evolved from a cyanobacteriophytochrome, to efficiently bind BV and brightly fluoresce in mammalian cells without the need for exogenous BV supply [40].

As the most red-shifted GFP-like FPs emit below 650 nm, near infrared (NIR) FPs have opened new opportunities for in vivo imaging, especially for deep-tissue and whole-body imaging, benefiting from the higher transparency, lower light scattering, and lower autofluorescence of mammalian tissues in the far-red and infrared region [41]. As an example, smURFP allowed imaging of HT1080 tumor xenografts in mice, without the addition of exogenous BV [38].

NIR FPs were also useful for the design of biosensors for the detection of protein–protein interactions or protease activity [42]. A split version of iRFP was obtained by separating the two distinct PAS and GAF domains [43]. Spatial proximity induces complementation of the two domains and thus iRFP recovers fluorescence upon BV binding. Split iRFP allowed for the study of protein–protein interactions in mammalian cells and in tumor xenograft in living mice. A reversible split version of IFP1.4 and monomeric split reporters of miRFPs were also designed to study protein–protein interactions in mammalian cells or in vivo models [36,43]. Infrared fluorescent proteins were also used to design protease biosensors. Proteases activity, especially caspases, induces proteolytic cleavage, leading to programmed cell death. The study of proteases activity can be useful to monitor apoptosis phenomena. The re-engineering of IFP allowed for the development of an infrared fluorogenic protease reporter, named iProtease, which becomes fluorescent upon protease activation. This biosensor uses a circular permutated version in which the native N and C termini are linked by a protease cleavage sequence. In this version, a cysteine essential for chromophore incorporation is physically displaced from the binding pocket, thus avoiding BV attachment and consequently fluorescence. Proteolytic cleavage induces IFP reformation, and the displaced cysteine return to the binding cavity, allowing for fluorescence in the presence of BV [44]. In addition, iCasper reporters activated by caspases 3 and 7 were also generated for the visualization of apoptosis during morphogenesis in *Drosophila* larvae and during tumorigenesis in the brain of *Drosophila*.

## 3. Fluorescent Reporters and Biosensors Based on Synthetic Fluorogenic Chromophores

### 3.1. Protein-Based Reporters

In 1998, Roger Tsien introduced the tetracysteine tag (CCXXCC) for the selective labeling of recombinant proteins in living cells [45,46,47,48]. This so-called self-labeling tag, which has a minimal size that limits perturbations of protein function, expression, and dynamics, selectively binds biarsenical derivates of fluorescein and resorufin, respectively, called FlAsH and ReAsH [45]. Upon covalent binding, FlAsH and ReAsH undergo some fluorescence activation. Because of this fluorogenic response, the tetracysteine tag/FlAsH system can be seen as one of the ancestors of more recent fluorogenic hybrid reporters.

The first genetically encoded protein tags activating the fluorescence of synthetic fluorogens upon recognition were human single-chain antibodies evolved to noncovalently recognize thiazole orange (TO) and malachite green (MG) derivatives [49]. Molecular recognition reduces the conformational flexibility of these molecular rotors, unquenching their fluorescence. Molecular engineering allowed for the expansion of the spectral properties of these fluorogen-activating proteins (FAP) from the blue to the far-red edge of the visible spectrum [50,51]. In their original version, FAPs required nonreducing environments (e.g., cell surface and secretory pathway) for the formation of disulfide bonds essential for proper functioning. The labeling of cytosolic proteins was rendered possible by the engineering of disulfide-free FAPs recognizing cell permeant MG-ester [52,53]. On the other hand, selective labeling of cell-surface proteins thanks to membrane-impermeant charged fluorogenic dyes opened the door to new ways to study membrane protein trafficking [54,55,56,57]. In this context, FAP has been used to study GABA receptor trafficking in neurons [58]. 

FAPs were used in various other fields of application. FAPs were shown to display a great potential for live-cell super resolution microscopy (e.g., stimulated emission depletion (STED)) because of the reduced photobleaching provided by the continuous fluorogen refreshment, and also for single molecule tracking in living cells, as only a subpopulation of proteins can be labeled using low fluorogen concentrations [59,60,61]. The FAP technology has also allowed for the design of photosensitizer molecules able to produce ROS upon illumination. In that case, FAPs bind heavy atom-substituted fluorogenic MG, which produce singlet oxygen upon near-infrared activation. This system, called FAP-Targeted and Activated Photosensitizer (TAPs), enabled cell ablation and subcellular protein inactivation [62]. This approach relies on the on-demand addition of fluorogens to the targeted cells. The near-infrared excitation and emission of FAP-based photosensitizers allows protein inactivation and cellular ablation in whole organisms. FAP-based photosensitizers allowed the CALI of proteins, targeted cell killing in cultured cells, and targeted lineage ablation in zebrafish (larva or adult) [62]. Apart from these developments, FAPs have been applied to in vivo studies for the detection of EGFR-enriched tumors in mice. A tumor-targeting system was developed by fusing a FAP to an affibody that specifically binds EGFR receptor. The resulting system, called affiFAP enabled tumor detection and can be useful for diagnosis, tumor margin definition, and surgery [63]. Finally, FAPs have been conjugated with immunoglobulin (IgGs) in order to label cell-surface proteins. This FAP-antibody tool was used to label cell-surface antigen, induce cell ablation of the antigen-expressing cells, and observe cell–cell contacts [64].

Another way to developed fluorogenic systems is to take advantage of the existing site-specific self-labeling systems, SNAP-tag, CLIP-tag, and Halo-tag [65,66,67], which allow the covalent labeling of proteins with chemical probes. SNAP-tag and CLIP-tag were evolved from the human DNA repair *O*^6^-alkylguanine-DNA alkyltransferase (AGT) [65], while Halo-tag was evolved from a bacterial dehalogenase [67]. SNAP-tag is a 20 kDa AGT variant that is capable of transferring the functionalized benzyl group of *O*^6^-benzylguanine (BG) derivatives to its active-site cysteine, leading to irreversible covalent labeling [65]. In CLIP-tag, a SNAP tag variant, the substrate is *O*^2^-benzylcytosine (BC) instead of BG [66]. Halo tag is a 33 kDa protein that is able to react with chloroalkane ligands [67]. As SNAP-tag, CLIP-tag, and Halo-tag substrates can bear various chemical probes, fluorogenic chromophores were used instead of regular fluorophores, so that the washing steps required to remove the remaining background signal from unreacted fluorophores was no longer necessary, resulting in higher contrast and higher temporal resolution imaging. Membrane-permeant near-infrared silicon-rhodamine (SiR) fluorophores have been used for the labeling of SNAP-tag, CLIP-tag, and Halo-tag [68]. Their fluorogenic properties rely on the zwitterion–spirolactone equilibrium of SiRs. The spirolactone (predominant in aqueous solution) is nonfluorescent, while the open zwitterion (favored in the less-polar protein proximity) displays excitation and emission maxima in the far-red and near-infrared. SiR fluorophores have been shown to be well suited for protein labeling in cells and tissues, multicolor imaging, and super-resolution imaging, including Stochastic optical reconstruction microscopy (STORM) and STED [68].

SiR analogues (Janelia fluorogens) with increased photostability and brightness were developed by replacing the N,N-dimethylamino group with a four-membered azetidine ring [69]. Fluorogens with various spectral properties were developed by additional molecular engineering [70]. The high cell permeability and fluorogenicity associated with these fluorogens allowed multicolor imaging in cells, tissues, and whole animals. As an example, Janelia probes have been used to image brain tissue from *Drosophila* larvae and mice [70]. In 2019, Lavis et al. showed that the equilibrium constant between the nonfluorescent spirolactone form and the zwitterionic fluorescent form can be exploited to predict fluorogenicity and could be useful for rational design [71]. In the same study, JF526 imaging with SNAP and Halo-tag proved to be well suited for multicolor super resolution microscopy (e.g., STED). In addition, JF526 can be easily modified into its hydroxymethyl derivative, which displays spontaneous blinking that can be used for single-molecule localization microscopy (SMLM) [71].

In order to study protein–protein interactions in living cells, a split SNAP protein complementation assay was developed. SNAP-tag protein was separated into two fragments by cleavage between amino acid residues 91 and 92 [72]. This split reporter was exploited to study the interaction between SpoIIQ and SpoIIIAH in sporulation of *Clostridium difficile* and also to demonstrate that Src kinases function as a dimer in living cells [73,74].

Very recently, a new chromophore series suitable for SNAP-tag derives from the linear merocyanine dye with an additional phenyl substituent was developed. The resulting near-infrared fluorophore named P-Mero4 displays a bathochromic shift (45 nm) due to unusual S-*cis* diene conformation [75]. Fluorescence increase in constrained environment results from limited phenyl rotation. Live-cell and in vivo imaging, using P-Mero4, was successfully achieved.

As the relatively large size of the self-labeling tags remains a concern for studies in cell biology, smaller protein tags have been developed. An example of a smaller tagging system is the photoactive yellow protein PYP, a small size protein (14 kDa) that has been used as covalent self-labeling system [76]. This protein, naturally found in *Halorhodospira halophila*, is a monomeric blue-light photoreceptor whose light-sensing ability comes from a hydroxycinnamoyl chromophore covalently attached to a cysteine residue via a thioester bond. Moreover, apoPYP was shown to form covalent thioesters with various coumarin derivatives. Fluorogenic labeling of PYP relies on either environmentally sensitive dyes depending on polarity, or on unquenching mechanisms [76,77,78,79]. Additional engineering of PYP-tag to control electrostatic interactions has provided an improved fluorogenic system for labeling intracellular proteins [80]. Furthermore, the incorporation of a hydroxyl group into environment-sensitive 7-dimethylamino-6-hydroxycoumarin generated an improved coumarin ligand (also called CG2), displaying higher reaction kinetics and optimal fluorescence activation. This improved ligand has been used to investigate the localization of sirtuin 3 (SIRT3), a mitochondrial deacetylase [81]. PYP labeling was recently used to develop probes for the quantitative and real-time imaging of methylated DNA [82]. The PYP-tagged methylated-DNA-binding domain was labeled with a DNA-binding fluorogen lighting up upon DNA recognition, which enabled the visualization of DNA methylation in living cells. 

Directed evolution of PYP recently allowed the development of fluorescence-activating and absorption-shifting tag (FAST) [83]. FAST was evolved to bind highly permeant hydroxybenzylidene rhodanine (HBR) analogues in a noncovalent manner. HBR analogues display a push–pull structure composed of an electron-donating phenol ring conjugated with an electron-withdrawing rhodanine heterocycle. These fluorogens dissipate light energy non-radiatively in solution, but strongly fluoresce when locked in a planar conformation within FAST. In addition, HBR analogs undergo an absorption red shift upon binding, which further increases their fluorogenicity as unbound fluorogens do not absorb at the wavelength used for exciting the emissive complex. Molecular engineering of the fluorogen structure has permitted the extension of the fluorescence emission color from green–yellow to the orange and red regions, allowing multicolor imaging [84,85]. Moreover, HBR analogs which are not able to cross the plasma membrane were developed for the selective labeling of membrane proteins at the cell surface and the study of their trafficking [86].

Using highly permeant HBR analogues, this fluorogenic system allowed the labeling of proteins in various localizations in living cells, such as bacteria, yeast, and mammalian cells, and in multicellular organisms such as zebrafish embryos, making FAST very promising for various in vivo applications [83]. In addition, as the fluorescence of FAST is completely oxygen-independent, it can be used in anaerobic conditions. Recently, this feature enabled the efficient labeling of proteins in strict anaerobes, such as *Clostridium* organisms, and the study of bacterial biofilm dynamics in low-oxygen environments [87,88]. 

Interestingly, this system is fluorescent instantaneously after folding, due to fast binding kinetics, allowing near-real-time experiments. Furthermore, the rapid exchange dynamics lead to an efficient fluorogen renewal, which reduces the apparent photobleaching rate [89]. The high dissociation rate constant makes FAST a highly dynamic and fully reversible noncovalent labeling system. FAST can be used as an ON/OFF switch: While the addition of fluorogen leads to an almost instantaneous labeling, the removal of the fluorogen by washing switches off the fluorescence and reverses labeling within a few seconds. By using fluorogens with different colors, this property allows for the swapping of color dynamically by fluorogen exchange. The unique kinetic signature of this color exchange was exploited to selectively detect FAST-tagged proteins in cells already tagged with both green and red reporters through two-color cross-correlation analysis [84]. Finally, FAST was shown to be suitable for SMLM and for Super-Resolution Radial Fluctuations (SRRF) microscopy for imaging sub-diffraction limited structures [90,91]. 

Split and circularly permutated versions of FAST were designed recently and used to study protein–protein interactions in real time and to develop biosensors [92,93]. Circularly permuted (cp)FAST was developed to design a calcium biosensor. This calcium intensiometric biosensor is based on the Ca^2+^-dependent interaction of calmodulin and the M13 peptide. The N-terminus and C-terminus of cpFAST were fused to the M13 peptide and to calmodulin, respectively. Calcium binding induces an increase of the fluorogen-binding affinity, leading to fluorescence activation. The biosensor color could be easily switched from green–yellow to orange–red by choosing the appropriate fluorogen [93]. A split fluorescent reporter with rapid and reversible complementation was recently designed by splitting FAST into two complementary fragments. This so-called splitFAST was shown to be suitable to study the dynamics of PPIs and to design biosensors (e.g., calcium and apoptosis) [92]. These studies show that cpFAST and splitFAST are promising modules for the design of various biosensors.

Other protein scaffolds were used for generating small protein tags. The 15 kDa cellular retinoic acid binding protein II (CRABPII) was engineered to form a covalent adduct with a nonfluorescent merocyanine dye precursor [94]. The generated protonated iminium forms a far-red fluorophore. Improved live-cell imaging was obtained by using human cellular retinol-binding protein II (hCRBPII)/merocyanine complex. This latter system displays a high quantum yield, making it brighter than most common red fluorescent proteins, and has been proven to be well suited for live-cell imaging in both cancer cell lines and yeast cells [95]. Recently, mutants of hCRBPII able to bind fluorogenic julolidine retinal analogue as an iminium were also engineered for the design of a ratiometric pH biosensor [96]. In this case, pH sensitivity relies on the presence of titratable amino acids side chain in the binding pocket. Through pH variation, electrostatic potential changes in the binding pocket, inducing a shift in the absorption/emission spectra of the julolidine retinal analogue. The pH is read, exciting the acid and basic forms at two distinct wavelengths. Apart from these examples, small fluorogen–protein systems have been developed from lipocalins [97,98]. These transport proteins are able to bind reversibly to various small-molecule ligands. To identify new fluorogen–protein pairs, in silico mutagenesis of amino acids within the ligand-binding pocket has been achieved. Lipocalin-based systems display high photostability and have been shown to be well suited for SMLM, using the fluorogenic chromophores named DiB1, DiB2, and DiB3. Very recently, a fluorogenic protein was designed de novo by using computational approaches, opening up new possibilities for the design of reporters and biosensors [99].

### 3.2. RNA-Based Reporters

The concept of fluorogen-activating tags was extended to other biomolecules such as RNA. RNA aptamers able to bind and activate the fluorescence of fluorogenic chromophores were generated by using systematic evolution of ligands by exponential enrichment (SELEX). A landmark study is the development of Spinach, an RNA aptamer that interacts noncovalently with 3,5-difluoro-4-hydroxybenzylideneimidazolidinone (DFHBI), an analog of the GFP chromophore 4-hydroxybenzylidene imidazolidinone (HBI) known to be fluorescent only within the *beta*-barrel of GFP [100,101]. Conformational locking of DFHBI upon binding to Spinach aptamer leads similarly to a drastic increase of the fluorescence, allowing for high-contrast imaging [102,103]. Further optimizations led to Spinach 2, designed to overcome misfolding issues encountered with the initial version [104], and Broccoli, an RNA aptamer optimized for cellular expression and intracellular imaging [105]. 

The color palette available for RNA labeling was further extended to the red edge of the visible spectrum, with the RNA aptamer Mango, which binds thiazole orange (TO) derivatives with nanomolar affinity [106] and, more recently, with the RNA aptamers, Corn, Orange Broccoli, and Red Broccoli, which bind 3,5-difluoro-4-hydroxybenzylidene-imidazolinone-2-oxime (DFHO), a fluorogen similar to the chromophore of red fluorescent proteins (RFP) [107], and with the aptamer Chili that binds 3,5-dimethoxy-substituted HBI (DMHBI) derivatives and exhibits a large Stokes shift [108]. The high photostability of Corn-DFHO has been used for the quantitative fluorescence imaging of mTOR-dependent Pol III transcription [107]. Recently, near-infrared RNA aptamers binding fluorogenic silicon rhodamines (SiRs) were identified. These are highly photostable and compatible with super-resolution microscopy techniques, such as live-cell STED imaging. Fluorogenicity here depends on the intramolecular spirocyclization of SiRs: the emission of fluorescence occurs only when SiRs are in the zwitterionic form within the RNA aptamer [109]. More recently, a new series of fluorogen-RNA aptamers called Pepper was developed. The associated fluorogen HBC (4-((2-hydroxyethyl)(methyl)amino)-benzylidene)-cyanophenylacetonitrile is nonfluorescent in solution but highly fluorescent in constrain environment. Various versions of Pepper ligands have been developed by modifying HBC structure, allowing to it to cover a large range of visible light spectrum, from cyan to red: Pepper 485 to 620 (corresponding to the maxima of emission) [110]. The Pepper system proved to be useful to study the spatiotemporal distributions of intracellular noncoding RNAs and mRNAs and to label endogenous chromosomal loci. Furthermore, Pepper 620, displaying high brightness and photostability, was shown to be well suited for live-cell imaging of RNA aptamers beyond the diffraction limit.

A new concept of fluorogenic aptamer was recently proposed with the design of a new bright and photostable fluorogen for RNA imaging relying on quenching mechanism. The resulting cell-permeable fluorogenic dimer sulforhodamine B dyes (Gemini-561) and the corresponding dimerized aptamer (o-Coral) allowed direct fluorescence imaging of RNA transcripts in live mammalian cells, without tag multimerization [111].

Apart from the study of the localization, transcription, and translation of RNA in cells, RNA aptamers can be used to study specific RNA. For example, miRNA expression sensors were recently developed. Small noncoding RNA involved in post-transcriptional regulation in living cells has been monitored by using Mango aptamer [112]. In addition, thanks to their small size, fluorogenic aptamers might be efficient tools to investigate the dynamic of viral RNAs [113,114]. For example, Spinach and Spinach2 aptamer have been successfully fused to HIV and to Sindbis virus genome, respectively. These studies show that aptamers can be useful to quantify and study the expression, distribution, and spread from cell to cell of viral RNA [113].

Biosensors able to sense various biomolecules, such as nitrogenous bases or cAMP, were developed by coupling Spinach and its derivatives to aptameric sensing units [107,115,116,117,118,119]. Broccoli has also been used to monitor the activity of RNA-modifying enzymes. Methylated Broccoli cannot bind its cognate fluorophore, and so is nonfluorescent. Thus, the activity of RNA demethylase restores the fluorescence of Broccoli, allowing the identification of the inhibitors of such enzymes [120]. 

## 4. Conclusions

The discovery and subsequent development of GFP-like proteins have constituted a major achievement in bioimaging. However, despite all their advantages, their size and tendency to oligomerize, their need for molecular oxygen, their slow fluorophore maturation, and their restriction to a genetically encoded fluorophore have been shown to limit their use [1,5,6]. In this context, the development in the last years of fluorogenic systems made of a genetically encoded domain (protein or RNA) able to bind and activate the fluorescence of fluorogenic chromophores has opened new exciting opportunities for the labeling of biomolecules in living systems.

The advantages offered by fluorogenic systems have been exploited in various applications. First, the oxygen-independency feature of some of these systems, such as FbFPs, UnaG, and FAST, was shown to allow the investigation of anaerobic organisms. This property opens new opportunities for exploring medically important organisms and microbial communities and, more generally, anaerobic biological phenomena in bacteria, yeast, mammalian, and plant systems [13].

Second, the development of NIR fluorescent probes has made possible deep-tissue and whole-body imaging. NIR fluorescent proteins, NIR FAP, or Janelia fluorogens were successfully used for in vivo labeling. In the future, such reporters should become essential tools for the in vivo monitoring of pathologies or the testing of therapeutic agents in animal models [121].

Third, several fluorogenic systems, notably based on self-labeling tag, FAP, and FAST have been demonstrated to be suitable for super-resolution imaging (such as STED, SMLM, or dSTORM) and single protein tracking. There is no doubt that, in the near future, new microscopy methods with improved spatial and temporal resolution will be developed, which will require engineering of fluorogenic systems with new or optimal properties.

Fourth, two systems, namely miniSOG and FAP-based photo-sensitizer, are able to generate ROS in addition to being fluorescent. This specific feature allows effective local photooxidation, cell ablation, or chromophore-assisted light inactivation (CALI), which opens new opportunities for biological studies. The development of the NIR FAP-based photosensitizer is a plus, as the blue light used for photosensitizing MiniSOG does not propagate efficiently within deep tissues and thus complicates in vivo experiments [122].

Fifth, fluorogen-based reporters allowed the creation of a vast collection of biosensors able to sense pH, oxygen, calcium, apoptosis, or protein–protein interactions relying eventually on modified (circularly permuted or split) reporters. Almost all the fluorogenic systems described above have been employed for biosensor design (FbFPs, UnaG, infrared FP, SNAP tag, FAST, CRABPII, etc.). In the future, biosensors that are engineered to be suitable for deep-tissue and whole-organism imaging could be valuable additions to the current fluorescence toolbox [123].

Last, the concept of fluorogen-activating tag was shown to be extendable to biomolecules other than proteins. In particular, the recent development of a collection of fluorogen-activating RNA aptamers opens exciting prospects for the observation and study of various RNAs, such as messenger RNA, micro RNA, viral RNA, or long noncoding RNA.

In this review, we distinguished reporters based on fluorogens from natural or synthetic origins. Each class has its pros and cons. The clear advantage of fluorogens being endogenously present in cells is that there is no delivery issue. However, the risk of hijacking natural fluorogen from its physiological function is to perturbate cellular processes and induce stress in living systems. Regarding synthetic fluorogens, the obvious advantage is that spectral and photophysical properties can be tailored by molecular engineering. In addition, fluorogen concentration can be adjusted, allowing on-demand applications (at a specific time or at a given intensity). Yet, as the chromophore is externally added, the latter needs to fulfill certain requirements, such as being nontoxic and membrane-permeant and not perturb cellular homeostasis. It is important to mention that there are no boundaries between natural and synthetic fluorogens. Indeed, natural chromophore properties can be improved by chemical synthesis. This is the case for biliverdin dimethyl esters, which display greater cell permeability than natural biliverdin, thus optimizing their use in living cells [39]. Reciprocally, it could be interesting to evolve tags (such as RNA aptamers) able to bind and activate the fluorescence of endogenous fluorogens.

The presented systems vary also by the way the fluorogen is bound to its cognate tag, whether it is bound covalently or noncovalently. Each class displays benefits. Covalent binding allows pulse-chase labeling to study protein synthesis, trafficking, and turnover. Moreover, high-contrast imaging can be obtained by washing away the excess of fluorogenic probes to further increase contrast. On the other hand, noncovalent systems enable more reliable real-time labeling, as the activation of fluorescence is, in general, faster, since no covalent bond has to be created. Moreover, it is possible to reverse the labeling by washing, opening new ways to control the fluorescence state of a reporter, and providing new opportunities for multiplexing. In addition, noncovalent fluorogen binding constitutes a great advantage for the design of reversible split systems and dynamic biosensors.

In conclusion, fluorogen-based reporters are very attractive alternatives to traditional GFP-like fluorescent proteins. They allow more and more sophisticated and challenging observations in living cells and organisms, and they open exciting new possibilities for whole-body imaging, biosensor design, multiplex imaging, and high-resolution imaging. The design and use of fluorogen-based reporters and biosensors are still in their infancy, and it is a safe bet to say that the coming years will see demonstrations of their full potentials and further exciting and unpredictable advances.
